# Exploiting pathogens' tricks of the trade for engineering of plant disease resistance: challenges and opportunities

**DOI:** 10.1111/1751-7915.12017

**Published:** 2013-01-02

**Authors:** Murray R Grant, Kemal Kazan, John M Manners

**Affiliations:** 1 College of Life and Environmental Sciences, University of Exeter, ExeterStocker Road, Exeter, EX4 4QD, UK; 2 CSIRO Plant Industry, Queensland Bioscience PrecinctBrisbane, Qld, 4069, Australia

## Abstract

With expansion of our understanding of pathogen effector strategies and the multiplicity of their host targets, it is becoming evident that novel approaches to engineering broad-spectrum resistance need to be deployed. The increasing availability of high temporal gene expression data of a range of plant–microbe interactions enables the judicious choices of promoters to fine-tune timing and magnitude of expression under specified stress conditions. We can therefore contemplate engineering a range of transgenic lines designed to interfere with pathogen virulence strategies that target plant hormone signalling or deploy specific disease resistance genes. An advantage of such an approach is that hormonal signalling is generic so if this strategy is effective, it can be easily implemented in a range of crop species. Additionally, multiple re-wired lines can be crossed to develop more effective responses to pathogens.

## Introduction

Recent efforts in sequencing of pathogen genomes have revealed numerous new insights into the processes employed by plant pathogens. One of such insights was the identification of surprisingly large numbers of candidate effector proteins encoded by pathogen genomes. We still have substantial progress to make in understanding how pathogen effectors would work. However, emerging evidence suggests that a remarkably diverse range of plant processes can be potentially targeted by these effectors. Identifying the structure of the molecular networks underpinning the two key plant defence processes; effector triggered immunity (ETI) and pathogen-associated molecular pattern (PAMP) triggered immunity (PTI) would require substantial new efforts. Despite this, recent insights into pathogen effector function provide new foundations for revisiting and reshaping biotechnological approaches to crop protection. This review will address our current understanding of pathogen infection processes from a global perspective, drawing on a limited selection of key examples of the defence networks targeted by pathogens to illustrate both the complexity and underlying communality in pathogen virulence strategies. We briefly examine opportunities and challenges in genetic-based disease intervention strategies and discuss the possibility of solutions that precisely target a universal pathogen virulence strategy, i.e. modulation of plant hormone signalling networks. Specifically, we raise the following questions. First, can we engineer plants to overcome pathogen virulence strategies by targeted intervention of effector-mediated transcriptional reprogramming? And second, based upon a systems level understanding of plant hormone signalling during infection, can we intelligently design strategies to attenuate pathogen virulence and therefore develop a framework for generating broad-spectrum pathogen-resistant crops?

## A generalized molecular description of plant disease resistance

Concomitant with entry into the host either through stomata, wounds or via a specialized haustorial structure, the pathogen betrays its presence through surface-exposed pathogen/microbe-associated molecular patterns (P/MAMPs), such as fungal chitin, bacterial flagellin, peptidoglycans or lipopolysaccharides (LPS). These molecules activate specific plant pattern recognition receptor-like kinases (PRRs) (Jones and Dangl, 2006). PRRs, often of the leucine-rich repeat (LLR) or lysin-motif (LysM) domain class, function as part of an immune recognition complex that perceives PAMPs and the signal is then transduced to downstream components through a phosphorylation cascade, leading to activated basal defences (Greeff *et al*., 2012). Successful pathogens have effector complements evolved to suppress PTI. However, ETI, typically orchestrated through the activities of cytoplasmically localized R proteins of the NBS-LRR (nucleotide binding site-LRR) class, recognizes the physical or biochemical presence of one or more effectors, providing a robust, second layer of post-delivery protection. This co-evolutionary battle selects for pathogens with effector complements evolved to evade PTI and ETI recognition. In parallel, host R proteins evolve new recognition specificities leading to highly polymorphic repertoires of both effectors and R proteins.

### What defines a pathogen?

A pathogen has two key goals to achieve when invading a host; to initially disarm basal defence networks and subsequently to liberate nutrients required for its own sustenance and multiplication. To achieve this, pathogens have evolved a set of proteins and small-molecule virulence factors (e.g. toxins) to overcome plant defence. A significant proportion of current molecular plant pathology research focuses on the function of pathogen effectors and over the past decade notable inroads have been made into understanding their collective mechanisms of action. Pathogen effector complements are highly redundant and dispensable, but are also highly evolved to exploit adapted host defences. An elegant example is the phased delivery of smut fungus, *Ustilago maydis*, effector proteins into host cells during infection of maize. The expression of smut effector genes during infection is consistent with initial delivery of highly conserved set of effectors to establish host compatibility, and subsequently deployment of a more adapted effector set to modulate host metabolic processes necessary for organ-specific tumorigenesis (Skibbe *et al*., 2010).

## Do we yet understand what makes a pathogen virulent?

There are many challenges to address before we fully understand pathogen virulence strategies, notwithstanding the interactions between effectors and the chemical activities of the small molecules they induce to promote disease. We still lack knowledge on the nature of the carbon and nitrogen compounds required for pathogen nutrition. One area that has received particularly little attention is role of host molecules that may specify the inductive signal for regulatory pathways activating effector cascades.

Whole-genome sequencing has provided an invaluable experimental resource with a plethora of pathogen genomes are now available (e.g. http://cpgr.plantbiology.msu.edu/). However, rather than simplifying our understanding, comparative genomics has revealed that pathogen effector complements are diverse and, depending upon the pathogen, either markedly reduced, such as in the pseudomonads, or expanded, most notably in oomycetes (Bozkurt *et al*., 2012). Indeed, ooymcete effector complements extend to many hundreds per species and exhibit little, if any, sequence homology although some structural similarity exists in the RxLR effectors (Boutemy *et al*., 2011; Win *et al*., 2012). While expansion is not so evident in fungal effector repertoires (Koeck *et al*., 2011; Rafiqi *et al*., 2012), comparative genomics indicates that extensive transposon-based genome expansion has occurred in powdery mildews despite little sequence homology existing between pea (*Erysiphe pisi*), barley (*Blumeria graminis*) and *Arabidopsis* (*Golovinomyces orontii*) powdery mildew isolates, indicative of strong selective adaptation (Spanu *et al*., 2010). The considerable expansion of retro-transposon derived repetitive DNA in the *Blumeria* genome appears characteristic of filamentous plant pathogens, particularly numerous *Phytophthora* isolates, where this genome plasticity is predicted to aid the emergence of new virulence traits (Raffaele and Kamoun, 2012).

Effector expansion is consistent with the complexity of these pathogens' lifestyles. The adoption of complex infection strategies, including haustorial establishment and maintenance appears to have led to a remarkable co-evolutionary adaptation of effector repertoires to specialized hosts. Despite the limited relatedness in primary sequence and effector complement, many taxonomically distinct pathogens share similar infection strategies and virulence mechanisms. Notably, bacterial pathogens, despite a much reduced yet still redundant effector repertoire, successfully cause disease on many of the same hosts that also support oomycete and fungal infections. Importantly, host defence regulatory hubs, such as EDS1 (enhanced disease susceptibility 1), NPR1 (nonexpresser of PR genes 1) and PAD4 (phytoalexin deficient 4), identified by genetic screens, are necessary for resistance to a range of diverse pathogens (Glazebrook, 2005). It is possible that these key defence components are desired targets of effectors from multiple pathogens (Mukhtar *et al*., 2011). For instance, *Arabidopsis* EDS1, which interacts with the TIR-NB-LRR (Toll-interleukin-1 receptor-nucleotide binding-leucine-rich repeat) class bacterial disease resistance proteins to initiate ETI, is targeted by multiple effectors (e.g. AvrRps4 and HopA1) of the bacterial pathogen *Pseudomonas syringae* (Bhattacharjee *et al*., 2011; Heidrich *et al*., 2011; see also below). It is therefore logical to conclude that plant defence signals converge on key host network components, which are necessary and essential to elaborate an effective immune response. Thus, a detailed knowledge of virulence mechanisms of a model pathogen will provide broad insight into the nature and diversity of general host signalling processes targeted during disease progression.

## *Pseudomonas syringae*; study of a model pathogen illuminates general virulence mechanisms

**Figure 1:**
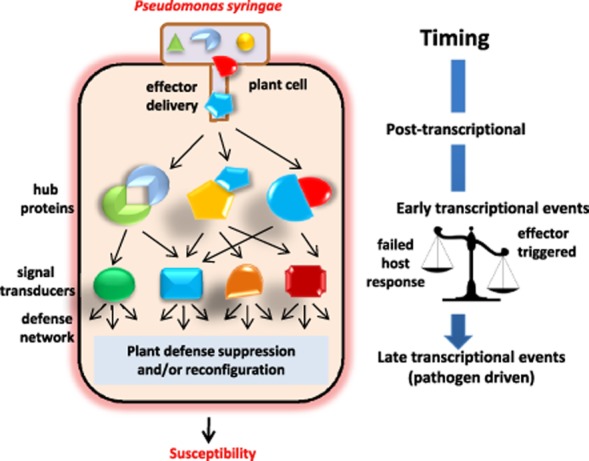
*Pseudomonas syringae* is used as an example to highlight pathogen virulence strategies during pathogen infection strategies and the timing of those events. The cartoon deciphers classical signalling networks engaged post-PTI, following activation of host pattern recognition receptors. *Pseudomonas syringae* pv. *tomato* DC3000 delivers 28 various effector proteins through the type III secretion system into the plant cell. These collaborate to target host proteins, directly or after post-delivery modifications, which may include phosphorylation, acetylation or proteolytic cleavage. Effector targets may include components of both ETI and PTI, shown as hub proteins. As effectors modulate host signalling pathways, there is a transcriptional reprogramming away from components underpinning activated basal defence towards induction of pathways that suppress basal defence, and later, reconfigure host metabolism for pathogen nutrition. This review proposes that judicious selection of unique, early host responsive promoters can be used to precisely control expression of re-engineered components of plant hormone response networks to nullify pathogen virulence strategies.

One of the best-studied plant pathogens molecularly is the hemi-biotrophic *P. syringae* pv. *tomato* DC3000 (DC3000), the causal agent of bacterial speck disease of tomato. DC3000 entry into plant via stomata or wounds triggers assembly of a functional type III secretion system (T3SS), encoded by *hrc*/*hrp* (*hypersensitive response conserved/hypersensitive response and pathogenicity*) genes which predominately reside in two main clusters; the conserved effector locus (CEL) containing universal and highly conserved effector genes and the exchangeable effector locus (EEL) containing more hypervariable determinants (Alfano and Collmer, 2004). DC3000 delivers 28 diverse and internally redundant effector proteins (Buell *et al*., 2003; Cunnac *et al*., 2009; 2011; Kvitko *et al*., 2009; Lindeberg *et al*., 2012) which variously contribute to suppression of plant defence and re-configuration of host metabolism for pathogen nutrition (Fig. 1).

Genomic sequencing of the three major pathovar clades represented by DC3000, *P. syringae* pv. *syringae* B728a (brown spot of bean) and *P. syringae* pv. *phaseolicola* 1448A (halo blight of bean) and numerous strains within each clade identified core effector sets (Joardar *et al*., 2005; Vencato *et al*., 2006; Vinatzer *et al*., 2006; Studholme *et al*., 2009; Baltrus *et al*., 2011) and a total of 57 effector families within the pangenome (Baltrus *et al*., 2011). Unexpectedly, these strains carry markedly different effector repertoires and *in silico* analyses provide no evidence for conservation of host-specific effectors. These comparative analyses did however, provide an intriguing insight into the impact of the host–pathogen co-evolutionary arms race, revealing the birth, death, migration and inactivation of various effectors.

Even within pathovar clades, diverse effector repertoires exist. Both DC3000 and *P. syringae* pv. *syringae* strain T1 cause bacterial speck disease on tomato yet share only 14 effectors (Almeida *et al*., 2009) highlighting the capacity of plant pathogenic pseudomonads to implement a range of virulence strategies. Interestingly, T1 carries a full-length HopAS1 effector and is non-pathogenic in non-host *Arabidopsis thaliana*, whereas all *P. syringae* strains carrying a truncated hopAS1 variant are pathogenic on *Arabidopsis*, indicating a single effector can contribute to restricting host range (Sohn *et al*., 2012).

Using *Nicotina benthamiana* as a host, elegant deconstruction and reassembly of the DC3000 effector repertoire by the Collmer lab demonstrated deletion of 15 DC3000 effectors had minimal effect on virulence but identified two essential redundant effector groups, comprising AvrPto/AvrPtoB(HopAB) and AvrE/HopM1/HopR1 (Kvitko *et al*., 2009; Cunnac *et al*., 2011), which target PAMP immune signalling and vesicle trafficking pathways respectively. Significant progress has been made in elucidating the function of many of these constituent effectors and the emerging paradigm is that the collective activities of these effectors paralyse plant defences by either physical inhibition, elimination or post-translational modification of host immunity proteins involved in PTI and ETI. Detailed descriptions of these effector activities are outside the scope of this review but are covered in the following excellent reviews (Deslandes and Rivas, 2012; Feng and Zhou, 2012; Lindeberg *et al*., 2012).

## Challenges in modifying host PTI and ETI networks

### The complexities in targeting PTI networks

Logic predicts targeting the apex of PAMP signalling would potentially provide broad-spectrum immunity. However, following remarkable progress in understanding effector activation and effector intervention in signalling from innate immune receptor complexes, particularly those involving the archetypal FLS2 (flagellin sensing 2) receptor, biotechnologically feasible solutions for enhancing host resistance are less obvious. Experimental evidence shows that the kinase domains of FLS2/EFR (elongation factor Tu PAMP receptor) and CERK1 (chitin elicitor receptor kinase 1) exist in pre-formed immune-receptor complexes constitutively interacting with BIK1 (Brassinosteroid Interacting Kinase 1). Perception of the MAMP ligands, elf18 or flg22, recruits the cytosolic BAK1 (Brassinosteroid Associated Kinase 1) and the resultant phosphorylation of BIK1's activation loop initiates downstream signalling cascades. An emerging theme is that core effector repertoires target components of the innate immune perception complex, including FLS2/CERK1/EFR/BIK1, and downstream phosphorylation cascade, using a variety of different strategies (see Deslandes and Rivas, 2012; Feng and Zhou, 2012 for recent reviews). Moreover, multiple redundant effectors from a single pathogen can act on PTI signalling networks, and many also target ETI processes, making re-engineering of PTI networks challenging. The evolved complexity of pathogen intervention in P/ETI is illustrated in the following two examples.

### HopAB – a multifunctional bacterial effector

The DC3000 effector HopAB (AvrPtoB) best encapsulates the evolved multifunctional complexity of effectors. HopAB encodes multiple activities in PTI and ETI, these activities being ascribed to both modular domains and the intact protein itself (He *et al*., 2006; Xiao *et al*., 2007; Shan *et al*., 2008; Xiang *et al*., 2008). The first 307 of 553 amino acids HopAB interact with the chitin binding LysM domain CERK1 to interfere with PTI signalling (Gimenez-Ibanez *et al*., 2009; Zeng *et al*., 2012). This domain also interacts with the tomato R protein kinase Pto to activate ETI (Gimenez-Ibanez *et al*., 2009). The N-terminal 137, but not 307 amino acids interact with (i) the kinase domain of FLS2 and BAK1 suppressing signalling following flagellin perception (Gohre *et al*., 2008; Shan *et al*., 2008) and (ii) with the Pto disease resistance homologue Fen (Rosebrock *et al*., 2007). The C-terminal RING finger and U-box E3 ligase domains participate in the proteasomal degradation of FLS2 and EFR, whereas full-length HopAB suppresses ETI by ubiquination of Fen, targeting it for degradation (Gohre *et al*., 2008).

### Multiple effectors camouflage *Cladosporium fulvum* from recognition by tomato

The fungal pathogen *Cladosporium fulvum* uses a complex multicomponent approach as one tactic to evade PTI. Chitin oligomers detected by PRRs are potent elicitors of PTI (Kaku *et al*., 2006; Shimizu *et al*., 2010). *Cladosporium fulvum* secretes Ecp6, a LysM chitin-binding domain protein, which selectively binds chitin oligosaccharides preventing recognition by tomato PRRs (Jonge *et al*., 2010). Moreover, *C. fulvum* secretes Avr4 apoplastically. Avr4 binds to the fungal wall chitin, preventing hydrolysis by tomato chitinase (Burg *et al*., 2006; Esse *et al*., 2007). *Cladosporium fulvum* also secretes Avr2, which binds and inhibits plant extracellular cysteine proteases required for basal defence (Esse *et al*., 2008). This collective assault on host defences means it is difficult for a host to overcome multiple effector activities targeted towards camouflaging the pathogen's presence, thus contributing to the pathogen durability.

## Strategies to overcome immune receptor intervention

### Deployment of PTI

Despite these challenges, Lacombe *et al*. exploited the finding that the EFR was evolutionarily constrained to the *Brassicaceae* (Lacombe *et al*., 2010), but that bacteria from diverse genera carried the highly conserved elf18 epitope of the ubiquitous EF-Tu protein recognized EFR. They explored interfamily transfer of EFR. Expression in *Solanaceae* plants *N. benthamiana* and tomato provided levels of enhanced resistance to a variety of bacterial pathogens with different lifestyles. Most notable, bacterial wilt conferred by the soil living *Ralstonia solanacearum* was dramatically attenuated in transgenic tomato expressing *A. thaliana* EFR (Lacombe *et al*., 2010). While promising, this strategy is restricted to identifying those PAMP receptors that, though host specialization, have evolved diverged PTI components.

## Combinatorial *R* gene approaches

Stacking *R* genes involved in recognizing the same specific range of pathogen isolates remains a core plant breeding strategy. The robustness of potential biotechnological approaches for R-Avr interactions was demonstrated by delivery of the *Hyaloperonospora parasitica* effector protein ATR13 with oomycete, bacterial and viral pathogens into a host carrying the cognate resistance protein RPP13, resulting in defence responses that are effective against all three pathogens (Rentel *et al*., 2008).

The caveat, however, is that *R* genes are extremely vulnerable to a single-loss of function mutations in corresponding *Avr* genes. Thus, superimposed upon their ability to target early events in PTI, pathogens also retain the inherent dispensability of the effector repertoire enabling the luxury of discarding ‘liability’ effectors without significant fitness costs, leading to the emergence of new pathogen races lacking the ability to elicit the signal(s) monitored by the cognate R protein(s). Indeed the majority of the currently defined *Avr* genes constitute part of the *P. syringae* ‘variable effector repertoire’ (Cunnac *et al*., 2009; 2011; Lindeberg *et al*., 2012) which undergo strong diversifying selection to avoid detection by the host. This mechanism is not restricted to bacterial pathogens. Multiple alleles of flax Avr456L carrying alterations of surface-exposed residues have been identified that evade direct recognition by the L resistance genes (Ellis *et al*., 2007; Koeck *et al*., 2011). In *H. parasitica* the ATR13 alleles show extreme levels of amino acid polymorphism, enabling them to evade recognition by the highly polymorphic RPP13 resistance protein in *Arabidopsis* (Allen *et al*., 2004).

## Convergence of virulence strategies

Despite the plethora of effectors deployed by a diverse range of pathogens, it is clear virulence strategies converge on common signalling pathways. A number of such loci, other than classical R proteins, representing core defence network components have been identified genetically. As alluded to above, mutations in key immune components such as EDS1, NPR1 and PAD4 lead to enhanced susceptibility to multiple pathogens with diverse infection strategies (Glazebrook, 2005). These proteins must represent components of a conserved branch of the plant defence network that integrates signals from activated immune complexes. Recently, EDS1 has been shown be involved in complex nuclear cytoplasmic signalling that involved its intimate association with multiple R proteins (RPS6, SNC1, RPS4 and other components such as the tetratricopeptide repeat protein SRFR1) (Bhattacharjee *et al*., 2011; Heidrich *et al*., 2011). Unfortunately, the potential to use these components for improved disease resistance may be limited, as they appear to integrate defence signals subsequent to upstream effector intervention.

Underpinning disease progression post suppression of basal defence is the requirement for the pathogen to extract nutritional resources from its host. Evidence that common mechanisms may be engaged to alter primary metabolism comes from the identification of SWEET genes (Chen *et al*., 2010; 2012). Sugar efflux specified by the SWEET class of hexose bidirectional transporters appears to be hijacked by effectors from both bacterial and fungal pathogens, despite their diverse lifestyles, to deliver carbohydrate apoplastically or via specialized haustorial feeding structures.

## Targeting hormones – a pre-emptive strike

As more and more functions are being transcribed to effectors, it is becoming increasingly clear that effectors can target multiple host proteins, which appear to function in unrelated pathways. Therefore, while there are many potential ways to intervene, pathogens have evolved multiple mechanisms to promote disease. Thus, a judicious approach is necessary to ensure broad-spectrum choices to outsmart pathogens. One potential strategy is to nullify effector modulation of host signalling networks downstream of overridden PTI and ETI defences. An emerging theme is that diverse pathogens hijack host hormone biosynthetic or signalling pathways to overcome innate immunity and reconfigure metabolic pathways for their nutrition. How effectors perturb hormonal signalling remain to be clarified but all evidence suggest hormonal perturbation underpins most, if not all, virulence strategies (Robert-Seilaniantz *et al*., 2011).

Recently it has been shown that pathogens hijack abscisic acid (ABA) signalling pathways to promote virulence, and this mechanism is shared by both necrotrophic and biotrophic pathogens (Asselbergh, De Vleesschauwer 0026; Hofte 2008; Ton *et al*., 2009). ABA biosynthetic mutants show reduced susceptibility to virulent *P. syringae*, whereas ABA accumulation compromised resistance to biotrophs and necrotrophs such as *Botrytis cinerea.* This strategy appears to be robust. For example, ectopic expression of HopAB induces *de novo* ABA biosynthesis (Torres-Zabala *et al*., 2007) but pathogen induced ABA is not attenuated following challenge with DC3000Δ*AvrPtoB*Δ*AvrPto* (M. Grant, unpublished), indicating multiple redundant pathways can result in enhanced ABA*.*

Other effectors have been shown to specifically modulate phytohormones to promote virulence. The *P. syringae* effector AvrB targets both PTI and ETI through phosphorylation of RIN4 (RPM1 interacting protein 4) and MPK4 (MAP kinase 4), which leads to the induction of JA response genes, classically associated with antagonism of SA signalling, and enhanced bacterial growth (He *et al*., 2004; Cui *et al*., 2010).

Here we propose that precise, temporally and spatially controlled modulation of pathogen induced hormonal changes could be an effective strategy to nullify pathogen virulence. One often overlooked role of phytohormones in defence is their potential regulation of phytoalexin production (see Grosskinsky *et al*., 2011; Ahuja *et al*., 2012 for recent reviews). Therefore, despite the species-specific nature of many phytoalexins, a major consequence of pathogen modulation of hormone pathways is likely to be restriction of phytoalexin production.

## Hormonal control of phytoalexins

The genetic and molecular revolution in plant molecular pathology in the late 1980s effectively overlooked a fundamental and critical aspect of plant defences, the exquisitely controlled induction of a plethora of plant antimicrobial defence compounds derived from secondary metabolism or of proteogenic origin. The latter include antimicrobial peptides, proteinase inhibitors, chitinases, glucanases and the archetypal Pathogenesis-Related 1 protein (PR1), whose function, despite being universally used as a marker of salicylic-based defence, remains unknown. The secondary metabolites are derived from pathways transcriptionally induced by PAMP receptor activation, including the shikimate (phenylpropanoids, stilbenes, terpenes), isopropenoids (diterpene and sequiterpene derivatives) and various alkaloid pathways.

Hormones appear to generically induce host-specific phytoalexins. For example, JA signalling largely influences camalexin production in *Arabidopsis* (Rowe *et al*., 2010), exogenous application of MeJA induced stilbene accumulation (Faurie *et al*., 2009) and in grapevine cell culture, the combined application of sucrose and MeJA stimulated the accumulation of trans-resveratrol and resveratrol glucosides (Belhadj *et al*., 2008). Ethylene and JA application collectively induced accumulation of maize kauralexins (Schmelz *et al*., 2011) and could also synergistically mimic *Fusarium graminearum* induced production zealexins, acidic sesquiterpenoid phytoalexin, in maize. Both these phytoalexins confer antifungal activity against numerous phytopathogenic fungi at physiologically relevant concentrations (Huffaker *et al*., 2011). Experiments specifically investigating the impact of hormone modulation on phytoalexin production are limited. Transgene induced cytokinin increases in tobacco led to SA and JA independent induced resistance to *P. syringae* pv. *tobacco*. Resistance was associated with the induction of the phytoalexins scopoletin and capsidiol, which could substitute *in planta* for the cytokinin signal (Grosskinsky *et al*., 2011; 2012).

In contrast, ABA negatively regulates the synthesis of elicitor-induced capsidiol in tobacco (Mialoundama *et al*., 2009) providing an elegant example of how ABA induced susceptibility can antagonize the key bioactives in plant defence.

## Alternative approaches to achieving robust immunity

### Re-wiring hormonal networks

Based upon both structural and predictive modelling it is now possible to contemplate targeting and neutralizing pathogen virulence strategies that antagonize hormone-regulated immune pathways. This will necessitate precision re-engineering of core pathogen-modulated hormone signalling pathway components and re-wiring these modified signalling component to a specific pathogen induced promoter that confers earlier and stronger temporal regulation than its cognate wild-type promoter. This approach relies upon the natural pathogen infection process to activate the interference strategy designed to attenuate pathogen virulence. To circumvent this strategy, the pathogen would need to reconfigure its own virulence programme with the collateral fitness costs.

Engineering a proactive response to locally increased hormone concentrations could be achieved by re-wiring hormone networks by deployment of promoters specifying highly localized, temporally and spatially controlled, precise responses to pathogens linked to re-engineered components of hormone signalling pathways. We will first consider promoter selection, then the possible re-engineering strategies.

#### Promoter selection

Specific temporal/spatial control is the key to re-wiring host signalling pathways to nullify pathogen virulence strategies. Transcriptional activation underpins pathogen virulence strategies, but occurs subsequent to PTI initiated phosphorylation cascades and post-delivery effector modifications. As yet, no systematic analyses of early effector responsive genes have been reported. Ideal candidate genes are those that are not PAMP responsive but are rapidly induced by virulent pathogens. In an on-going project (http://www2.warwick.ac.uk/fac/sci/lifesci/research/presta/) analysis of high-resolution time-course microarrays reporting *P. syringae* infection of *Arabidopsis* using the virulent DC3000 strain, a cognate *hrp* mutant and mock challenge has revealed a large range of promoter dynamics. Thus it is possible to identify examples of early (within 3 hpi) effector responsive genes whose low/undetectable basal transcript levels are induced well in excess of an order of magnitude, through to promoters specifying moderate expression levels which subsequently saturate the microarray. Candidate genes can be identified showing either sustained or transient induction that are not PAMP, drought, high-light, circadian or senescence responsive, providing a tightly controlled effector responsive transcriptional unit to drive re-wiring of plant defence response networks.

#### Re-wiring ABA signalling

Hijacking ABA signalling is a key pathogen virulence strategy (Anderson *et al*., 2004; Torres-Zabala *et al*., 2007; Grant and Jones, 2009; Robert-Seilaniantz *et al*., 2011). ABA biosynthetic mutants show reduced susceptibility to both biotrophs (e.g. *P. syringae*) and necrotrophs (e.g. *B. cinerea*)*.* Thus precision re-wiring ABA signalling at the apex of the network (ABA perception) offers potential for broad-spectrum resistance to both biotrophs and necrotrophs.

#### The ABA perception network

Upon ABA binding, cytosolic pyrabactin resistance 1 (PYR1)/PYR1-like (PYL)/regulatory components of ABA receptors (RCAR) ABA receptors interact with the active site of the negative regulators of ABA signalling, Clade A protein phosphatase 2Cs (PP2C) and inactivates them. In the absence of ABA, Clade A PP2Cs interact with and inhibit the sucrose non-fermenting related kinase (SNRK) kinases (Cutler *et al*., 2010; Weiner *et al*., 2010). The loss of function triple mutant (*snrk2.2*/*snrk2.3*/*snrk2.6*) is defective in all known ABA responses (Fujii and Zhu, 2009). Thus, ABA/PYL-induced PP2C inhibition depresses SnRK2s, activating downstream ABA signalling networks. Below we discuss possibilities of re-engineering the PLY receptors and PP2C-negative regulators to attenuate ABA induced virulence.

#### Re-engineering PP2Cs to attenuate ABA signalling

Mutation of the active site of selected Clade A PP2Cs (glycine to aspartic acid substitution) will disrupt PYL–PP2C interactions (Melcher *et al*., 2009; Miyazono *et al*., 2009) but retain the ability to dephosphorylate target SNRKs therefore inactivating ABA signalling at two key nodes.

#### Engineering PYL receptors to function as ABA ‘sponges’

Based upon the PYL/ABA/PP2C complex crystal structure predict it is possible to engineer high-affinity ABA receptors that can ‘mop up’ large quantities of pathogen induced ABA but not bind to and inactivate PP2Cs (Santiago *et al*., 2012).

#### Interfering with ABA biosynthesis

In a dual strategy that complements the PYL engineering, it is possible to abrogate pathogen induced ABA accumulation by re-wiring ABA 8′-hydroxylase (Umezawa *et al*., 2006) to a pathogen responsive promoter. Coupled to an effector regulated promoter this strategy will specifically catabolize pathogen induced ABA.

### Re-wiring JA response networks

JA signalling antagonizes SA signalling and a number of biotrophic pathogens exploit this property to attenuate host defences (Glazebrook, 2005). A striking example is the production of the phytotoxic polyketide coronatine by many *P. syringae* pathovars. This virulence factor is a structural mimic of the plant bioactive JA, 3R,7S-jasmonoyl-isoleucine (JA-Ile) (Fonseca *et al*., 2009a). Pathogen coronatine production interferes with functionally antagonistic SA and JA signalling networks to disrupt plant immune responses and confer a fitness advantage (Brooks *et al*., 2005; Laurie-Berry *et al*., 2006). The recently identified JAZ (jasmonate-ZIM domain-containing) proteins repress jasmonate (JA) responsive transcription factors, most notably AtMYC2, a key regulator of JA responses (Anderson *et al*., 2004; Lorenzo *et al*., 2004; Fonseca *et al*., 2009b). In the presence of coronatine, JAZs are ubiquitinated by the F-box component COI1 of the E3 ubiquitin ligase complex (SCF^COI1^) and subsequently degraded by the 26S proteasome, freeing AtMYC2 to activate JA signalling networks (Lorenzo and Solano, 2005; Chini *et al*., 2007; Thines *et al*., 2007; Melotto *et al*., 2008; Fonseca *et al*., 2009b).

#### Interfering with the jasmonate COI1 receptor

Differential splicing of *JAZs* can lead to the splice variants lacking the Jas domain PY motif resulting in enhanced resistant to ubiquitin mediated proteasomal degradation (Chung *et al*., 2010). To attenuate JA signalling and its attendant suppression of SA defences, one could envisage re-wiring engineered JAZ splice variants lacking the C-terminal Jas domain, thus generating a JAZ variant that is resistant to ubiquitin mediated proteasomal degradation. These modified JAZs would bind to and effectively ‘poison’ COI1, preventing other JA repressors binding, thus creating a dominant JA-insensitive phenotype and prevent pathogens exploiting JA signalling pathways through COI1.

## Revisiting the deployment of *R* genes in plant defence

The AvrBs3/PthA family of Transcription Activator Like (TAL) effectors are found in plant pathogenic *Xanthomonas* spp. and *R. solanacearum.* TALs contain an acidic activation domain, a C-terminal nuclear localization signal and a central domain containing a variable number of 34 amino acid repeat modules (Boch and Bonas, 2010; Bogdanove *et al*., 2010). These modules contain repeat variable diresidues (RVD) at positions 12 and 13. Crystallization of TAL revealed that these repeat modules fold into two nearly identical alpha helices connected by a loop formed by the RDV. The amino acid at position 12 stabilizes the loop between repeats whereas the amino acid at position 13 makes base-specific contact with the DNA sense strand (Mak *et al*., 2012).

Thus the ability design TALs that target specific genomic regions, and link this to functional domains such as nucleases holds huge biotechnological promise for precision/customizable engineering and synthetic biology (Bogdanove and Voytas, 2011). Of relevance to engineering broad-spectrum pathogen resistance, artificial TALEs could be constructed that recognize selected disease resistance gene promoters to activate ETI. These designer transcriptional activators could be driven by the same repertoire of promoters discussed above. Like any new technology caveats apply. Notably, spatial considerations appear to lead to RVDs having different strengths. These issues have been recently explored (Garg *et al*., 2012) and recommendation to construct reliably functional TALEs have been suggested (Streubel *et al*., 2012) and tools such as TAL Effector-Nucleotide Targeter (TALE-NT) are being developed for TAL effector design and target prediction (Doyle *et al*., 2012). Alternatively, selected crop *R* genes could be engineered with synthetic promoters which are activated by a small set of TALEs, providing added system robustness.

## Conclusion

The last 5 years has seen unprecedented progress in identifying function of pathogen effector molecules and publication of a plethora of pathogen genomes. While comparative genomics has revealed that pathogen effector repertoires reflect lifestyle and infection strategies, functional studies have provided evidence that effectors have multiple targets and are often functionally redundant, highlighting an emerging paradigm that pathogens implement a range of virulence strategies by hijacking various host signalling networks. Thus, re-engineering or remodelling specific host targets is unlikely to lead to durable resistance.

Emerging evidence suggests hormonal perturbation underpins most, if not all, phytopathogen virulence strategies. Pathogens first reconfigure the host transcriptome in a precise, temporally controlled manner. With major progress being achieved in understanding phytohormone perception and signalling pathways, it is an opportune time to consider wiring highly specific pathogen responsive promoters to re-engineered components of hormone signalling to target and nullify pathogen virulence strategies that antagonize hormone-regulated immune pathways. This approach relies upon the natural pathogen infection process to activate the interference strategy designed to attenuate pathogen virulence. To circumvent this strategy, the pathogen would need to reconfigure its own virulence programme with the collateral fitness costs. If successful, the generic nature of this approach means it can be implemented across a range of crop species, and lends itself to stacking multiple re-wired lines by crossing to enhance pathogen resistance.

Using judicious selection of promoters, a similar strategy deploying precise, temporally and spatially controlled modulation of engineered TAL effectors targeting selected *R* genes could be an alternative strategy to generate broad-spectrum resistance.

## Funding Information

Some ideas in this review have evolved from British Biotechnology and Science Research Council-funded Grant BB/F005903/1.
